# Association between sleep duration on workdays and kidney stone expulsion in US adults: insights from the national health and nutrition examination survey 2007–2020

**DOI:** 10.1016/j.pmedr.2025.103109

**Published:** 2025-05-16

**Authors:** Shuchao Ye, Dongming Lu, Damei Ye, Bingyong You, Yongyang Wu, Shangfan Liao

**Affiliations:** a Department of Urology, Affiliated Sanming First Hospital, Fujian Medical University, Sanming 365000, Fujian, China; b Department of Rheumatology Immunology, Affiliated Sanming First Hospital, Fujian Medical University, Sanming 365000, Fujian, China

**Keywords:** Sleep duration, Kidney stone, Kidney stone expulsion, National health and nutrition examination survey, Cross-Sectional Study

## Abstract

**Objective:**

This cross-sectional study aimed to investigate the association between weekdays/workdays sleep duration (SDW) and kidney stone expulsion (KSE) in adults with kidney stones.

**Methods:**

We utilized data from the National Health and Nutrition Examination Survey (NHANES) conducted from 2007 to March 2020 (pre-pandemic). Adults aged ≥20 years with confirmed kidney stones were included. SDW served as the primary exposure variable, while KSE was the outcome. Multivariable logistic regression and restricted cubic spline (RCS) regression were employed to explore the SDW-KSE relationship.

**Results:**

A total of 2,040 participants with KSE and 1,966 without KSE were analyzed. In both unadjusted and fully adjusted logistic regression models, SDW was significantly associated with a lower odds of KSE (OR: 0.81 [0.77, 0.84] and 0.80 [0.74, 0.86], respectively). RCS analysis showed a non-linear association between SDW and KSE (p = 0.01). In the fully adjusted model, the odds of KSE decreased sharply with SDW beyond seven hours (OR: 0.69 [0.59, 0.80]).

**Conclusions:**

In this nationally representative sample, longer SDW was associated with a reduced likelihood of KSE. However, given the cross-sectional nature of our study, this association does not imply causality. Further experimental and longitudinal research is needed to elucidate the causal pathways and underlying mechanisms linking SDW and KSE.

## Highlights

• Longer workdays sleep is linked to a lower rate of kidney stone expulsion in adults.
• Sleep duration beyond seven hours reduces the likelihood of kidney stone expulsion.
• Optimising sleep may help improve conservative management of kidney stones.

## 1 Introduction

Urinary stones, a prevalent condition of the urinary system, currently affect approximately 8.8 % of the population, a figure that has quadrupled over the past five decades (Zampini et al., 2019). The presence of urinary stones inflicts significant pain on millions globally and may elevate the risk of developing chronic kidney disease (CKD) (Chuang et al., 2020), which has profound implications for public health and quality of life. Numerous factors influence the formation and expulsion of urinary stones, including dietary and lifestyle choices, environmental climate, metabolic disorders, and genetic predispositions (Bazyar et al., 2019; Kuta et al., 2016; Hu et al., 2014; Gan et al., 2023). Current treatment options for urinary stones primarily include conservative management, extracorporeal shock wave lithotripsy, and various minimally invasive endoscopic surgeries (Shen et al., 2022). Nonetheless, the influence of sleep patterns on the conservative management of kidney stones has received limited attention and remains insufficiently characterized.

Recent investigations have underscored the potential significance of sleep patterns in the pathogenesis of kidney stones (Yin et al., 2022; He et al., 2023; Yan et al., 2024). Diminished sleep duration may extend periods of food consumption, thereby heightening energy intake, which can precipitate a cascade of metabolic disorders such as obesity and diabetes, ultimately elevating the risk of kidney stone formation (Taheri et al., 2004). Conversely, excessive sleep duration has been associated with adverse outcomes, including cardiovascular and cerebrovascular diseases, cancer, stroke, and cognitive decline (Jike et al., 2018). While extensive research has been conducted on sleep duration and its impact on various health outcomes, its correlation with kidney stone expulsion (KSE) remains inadequately explored in the existing literature.

Due to professional and social obligations, sleep patterns on weekdays often differ from those on weekends. Understanding how sleep duration on weekdays or workdays (SDW) affects kidney stone expulsion in adults with kidney stones could provide valuable insights into the prevention and management of kidney stones. Sleep duration may influence renal endocrine function, including hormonal regulation of water and electrolyte balance, which plays a critical role in stone formation and expulsion.

The National Health and Nutrition Examination Survey (NHANES) offers a unique opportunity to explore this connection due to its extensive, nationally representative sample and comprehensive data on sleep habits, health conditions, and lifestyle factors. The primary objective of this study was to determine whether sleep duration on weekdays/workdays is associated with kidney stone expulsion in adults with kidney stones.

## 2 Materials and methods

### 2.1 Study design

The National Center for Health Statistics (NCHS) conducts the NHANES, a survey designed to assess the health and nutritional status of the U.S. population. It employs a comprehensive, multi-stage, and hierarchical probability sampling method to select participants from across the United States, ensuring a nationally representative sample. NHANES encompasses a wide array of data collection techniques, including interviews, physical examinations, and laboratory tests, which gather extensive demographic information, sleep pattern data, and health status indicators. The primary goal of NHANES is to evaluate the overall health and nutrition of the U.S. population. All procedures for the NHANES programme received approval from the National Center for Health Statistics Ethics Review Board, and informed written consent was secured from each participant prior to data acquisition. As the NHANES database is de-identified and data are publicly accessible, the present secondary analysis was exempt from further institutional ethical review. Throughout the study, researchers had no access to personal identifiers or any information that could compromise participant confidentiality.

### 2.2 Participants

**Fig. 1:**
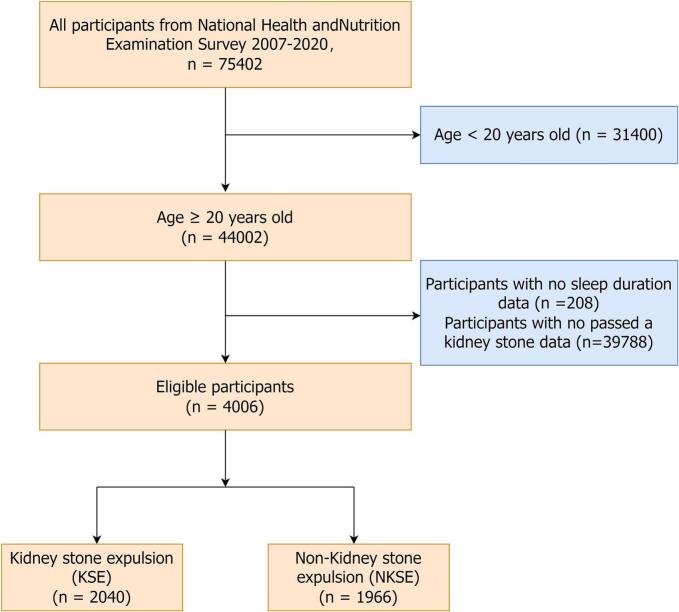
Flow chart of participant screening.

We utilized data from the NHANES cycles spanning from 2007 to March 2020 pre-pandemic (n = 75,402) (https://www.cdc.gov/nchs/nhanes), as the relevant data on kidney stones were only collected during this period. The research focused on individuals aged 20 years or older (n = 44,002). After excluding 208 participants with missing sleep duration data and 39,788 participants with missing data on kidney stone expulsion, 4,006 subjects were included in the final analysis. Fig. 1 illustrates a comprehensive flowchart outlining the process of recruiting study participants. Among the 4006 participants, the amount of missing values for the covariates were 1267 (31.63 %) for metabolic equivalent, 415 (10.36 %) for kilocalorie, 2 (0.05 %) for marital status, 4 (0.1 %) for educational level, 382 (9.54 %) for the ratio of family income to poverty, 758 (18.92 %) for alcohol, 2 (0.05 %) for smoke, 216 (5.39 %) for body mass index status, 15 (0.37 %) for diabetes mellitus, 29 (0.72 %) for coronary heart disease, 154 (3.84 %) for hyperlipidemia, 372 (9.29 %) for estimated glomerular filtration rate, 372 (9.29 %) for creatinine, 375 (9.36 %) for uric acid, and 377 (9.41 %)for calcium. There were no missing data for age group, sex, race, and hypertension.

### 2.3 Variables

#### 2.3.1 Outcome variable

The outcome variable was kidney stone expulsion (KSE) in adults with kidney stones. In the NHANES 2007–2014 cycle, participants who answered ≥1 to the question “How many times have you/has SP passed a kidney stone?” were classified as KSE, while those who answered zero were classified as none experiencing kidney stone expulsion (NKSE). In the NHANES 2015–2020 cycle, participants who answered “yes” to the question “In the past 12 months, have you/has SP passed a kidney stone?” were classified as KSE, while those who answered “no” were classified as NKSE..

#### 2.3.2 Primary exposure variable

The primary exposure variable was self-reported sleep duration on weekdays or workdays (SDW). Participants were asked questions such as “How much sleep do you/does SP usually get at night on weekdays or workdays?” or “Number of hours usually sleep on weekdays or workdays?” SDW was measured in half-hour increments, with <3 h recorded as two hours and ≥ 12 h recorded as 12 h. SDW was analyzed as a continuous variable and categorized into four groups: ≤5 h, >5 and ≤ 7 h, >7 and ≤ 9 h, and ≥ 9 h.

#### 2.3.3 Covariates

Demographic and socioeconomic characteristics included age (20-39, 40-59, 60-79, ≥80 years), sex, race, marital status, education level, and the ratio of family income to poverty (PIR).

Lifestyle habits include four variables, including smoking (never smoked means smoked less than 100 cigarettes in life. Former smoked means smoked more than 100 cigarettes in life and smoke not at all now. Now smoked means smoked moth than 100 cigarettes in life and smoke some days or every day), drinking(Rattan et al., 2022) (never drink means had <12 drinks in lifetime. Former drink means had ≥12 drinks in one year and did not drink last year, or did not drink last year but drank ≥12 drinks in lifetime. Mild drink means one drinks per day is for female and two drinks per day is for male. Moderate drink means ≥2 drinks per day for females, ≥3 drinks per day for males, or binge drinking ≥2 & <5 days per month. Heavy drink means ≥3 drinks per day for females, ≥4 drinks per day for males, or binge drinking [≥4 drinks on the same occasion for females, ≥5 drinks on the same occasion for males] on five or more days per month), physical activity (recorded as Metabolic equivalent (MET)), caloric intake (recorded as kilocalorie (KCAL)).

Physical measurements included body mass index (according to the WHO BMI classification(Obesity: Preventing and Managing the Global Epidemic, 2000) underweight was <18.5 kg/㎡, normal weight was 18.5 to <25 kg/㎡, overweight was 25 to <30 kg/㎡, and obesity was ≥30 kg/㎡). Additional conditions comprised hypertension, diabetes, coronary heart disease, hyperlipidemia, estimated glomerular filtration rate (eGFR), serum creatinine, uric acid, and calcium.

### 2.4 Statistical analysis

In the baseline tables, continuous variables are expressed as mean and standard deviation (SD), and intergroup comparisons were performed using t-tests. Categorical variables are summarized as frequencies and percentages [n (%)], with group differences evaluated by the chi-square test. We compared the baseline characteristics of participants in the final analysis according to KSE status. We also compared these characteristics with those participants who were excluded because of missing data on SDW or KSE.

Two logistic regression models were constructed: Model 1 (unadjusted); Model 2 (fully adjusted: age group, sex, race, metabolic equivalent, kilocalorie, marital status, educational level, the ratio of family income to poverty, alcohol, smoke, body mass index status, hypertension, diabetes mellitus, coronary heart disease, hyperlipidemia, estimated glomerular filtration rate, creatinine, uric acid, calcium).

To further verify the correlation between sleep duration and kidney stone excretion, sleep duration was treated as a categorical variable (≤5 h, >5 h and ≤ 7 h, >7 h and ≤ 9 h, ≥9 h), and the P value for trend was calculated. Tests for trend were performed by assigning the median value of each sleep duration category and including this as a continuous variable in the logistic regression analysis. Finally, restricted cubic spline (RCS) regression was used to explore the nonlinear relationship between SDW and KSE.

To reduce the bias that may be caused by missing values among covariates and to improve the statistical effect of accurately describing the target sample at the modelling stage, we performed multiple interpolations for the missing data to test the stability of the results.

All statistical analyses were performed using R version 4.4.1 (http://www.r-project.org), and statistical significance was set at P < 0.05. Data were analyzed using the survey weights recommended by NHANES and the sample was considered representative of the population.

## 3 Results

A total of 4,006 participants (2,040 participants who experienced kidney stone expulsion and 1,966 participants who never experienced kidney stone expulsion) were included in the study, representing 21,786,283 American adults. Compared with NKSE participants, KSE participants had lower SDW and MET, but higher KCAL. Lower age stratification (20–59 years), male sex, Non-Hispanic White ethnicity, marital status of married or living with a partner, lower education level, former drinking status, non-diabetes, and higher blood calcium levels were associated with a higher rate of KSE. Table 1 presents the baseline characteristics of participants according to their kidney stone expulsion status. There are significant differences in some baseline characteristics between the include group and excluded groups (Supplementary Table 1).

**Table 1:** Characteristics of participants by kidney stone expulsion status from the national health and nutrition examination survey dataset (2007–2020).

Variable	Total (n = 4006)	Kidney stone expulsion (n = 2040)	Non- Kidney stone expulsion (n = 1966)	P value
Sleep duration (hours), Mean ± SD	7.13 ± 1.65	6.85 ± 1.57	7.42 ± 1.68	<0.01^⁎^
Metabolic equivalent, Mean ± SD	5456.17 ± 7608.34	5099.97 ± 7263.95	5810.55 ± 7922.93	0.01^⁎^
Kilocalorie, Mean ± SD	3774.07 ± 1657.53	3829.30 ± 1649.80	3688.81 ± 1666.54	0.03^⁎^
Age group (years), n (%)				<0.01^#^
20-39	759(18.95)	431(21.13)	328(16.68)	
40-59	1357(33.87)	698(34.22)	659(33.52)	
60-79	1504(37.54)	737(36.13)	767(39.01)	
≥80	386(9.64)	174(8.53)	212(10.78)	
Sex, n (%)				0.03^#^
Female	1797(44.86)	880(43.14)	917(46.64)	
Male	2209(55.14)	1160(56.86)	1049(53.36)	
Race, n (%)				<0.01^#^
Non-Hispanic White	2084(52.02)	1152(56.47)	932(47.41)	
Non-Hispanic Black	574(14.33)	235(11.52)	339(17.24)	
Mexican American	491(12.26)	251(12.30)	240(12.21)	
Other Hispanic	467(11.66)	240(11.76)	227(11.55)	
Other Race - Including Multi-Racial	390(9.74)	162(7.94)	228(11.60)	
Marital status, n (%)				<0.01^#^
Never married	421(10.51)	207(10.15)	214(10.89)	
Divorced, separated, or widowed	1082(27.02)	506(24.82)	576(29.31)	
Married or living with partner	2501(62.46)	1326(65.03)	1175(59.80)	
Educational level, n (%)				0.01^#^
Below high school	978(24.44)	517(25.37)	461(23.47)	
High school	918(22.94)	495(24.29)	423(21.54)	
Above high school	2106(52.62)	1026(50.34)	1080(54.99)	
The ratio of family income to poverty, n (%)				0.12^#^
Low	1117(30.82)	608(32.34)	509(29.19)	
Medium	1417(39.10)	719(38.24)	698(40.02)	
High	1090(30.08)	553(29.41)	537(30.79)	
Alcohol, n (%)				<0.01^#^
never	434(13.36)	221(12.48)	213(14.42)	
former	559(17.21)	386(21.80)	173(11.71)	
mild	1283(39.50)	668(37.72)	615(41.64)	
moderate	453(13.95)	213(12.03)	240(16.25)	
heavy	519(15.98)	283(15.98)	236(15.98)	
Smoke, n (%)				0.49^#^
never	2012(50.25)	1006(49.34)	1006(51.20)	
former	1206(30.12)	628(30.80)	578(29.41)	
now	786(19.63)	405(19.86)	381(19.39)	
Body mass index status, n (%)				0.19^#^
Underweight	28(0.74)	17(0.87)	11(0.60)	
Normal	715(18.87)	365(18.74)	350(19.00)	
Overweight	1254(33.09)	671(34.45)	583(31.65)	
Obese	1793(47.31)	895(45.94)	898(48.75)	
Hypertension, n (%)				0.10^#^
no	1741(43.46)	913(44.75)	828(42.12)	
yes	2265(56.54)	1127(55.25)	1138(57.88)	
Diabetes mellitus, n (%)				<0.01^#^
no	2498(62.59)	1293(63.66)	1205(61.48)	
Impaired fasting glucose	181(4.54)	87(4.28)	94(4.80)	
Impaired glucose tolerance	99(2.48)	69(3.40)	30(1.53)	
yes	1213(30.39)	582(28.66)	631(32.19)	
Coronary heart disease, n (%)				0.05^#^
no	3655(91.90)	1884(92.76)	1771(91.01)	
yes	322(8.10)	147(7.24)	175(8.99)	
Hyperlipidemia, n (%)				0.07^#^
no	927(24.07)	452(22.84)	475(25.36)	
yes	2925(75.93)	1527(77.16)	1398(74.64)	
Estimated glomerular filtration rate (mL/min/1.73 m^2^), Mean ± SD	86.28 ± 24.23	86.60 ± 24.06	85.94 ± 24.40	0.41^⁎^
Creatinine (mg/dl), Mean ± SD	0.97 ± 0.64	0.97 ± 0.63	0.97 ± 0.65	1.00^⁎^
Uric acid (mg/dl), Mean ± SD	5.64 ± 1.52	5.68 ± 1.51	5.59 ± 1.52	0.07^⁎^
Calcium (mg/dl), Mean ± SD	9.34 ± 0.39	9.39 ± 0.41	9.30 ± 0.37	<0.01^⁎^

Data was expressed as Means ±SD or n (%). SD, standard deviation; n (%), frequencies (percentages). ⁎t-test; # chi-square.

In both unadjusted and fully adjusted logistic regression models (Table 2), SDW was significantly associated with a lower odds of KSE (OR: 0.81 [0.77, 0.84] and 0.80 [0.74, 0.86], respectively). This trend remained significant across different SDW groups (P < 0.01), and the negative correlation was primarily observed in participants whose SDW beyond seven hours. Importantly, the main results and the direction of the effect values of the two different logistic regression models after multiple interpolation of the missing values of the covariates are consistent with our initial analysis (Supplementary Table 2), underlining the robustness of our conclusions.

**Table 2:** Association of sleep duration on weekdays or workdays with kidney stone expulsion, national health and nutrition examination survey dataset (2007–2020).

Exposure	Model 1	Model 2
	OR (95 %CI)	OR (95 %CI)
Sleep duration	0.81 (0.77,0.84)	0.80 (0.74,0.86)
Sleep duration		
[2 h-5 h]	1.00	1.00
(5 h-7 h]	0.83 (0.68,1.01)	0.87 (0.62,1.23)
(7 h-9 h]	0.52 (0.42,0.63)	0.60 (0.42,0.87)
(9 h-12 h]	0.25 (0.19,0.34)	0.16 (0.09,0.31)
P for trend	<0.01	<0.01

P value was calculated by logistic regression. OR, odds ratio; CI, confidence interval; h, hours. Model 1 (unadjusted); Model 2 (fully adjusted: age group, sex, race, metabolic equivalent, kilocalorie, marital status, educational level, the ratio of family income to poverty, alcohol, smoke, body mass index status, hypertension, diabetes mellitus, coronary heart disease, hyperlipidemia, estimated glomerular filtration rate, creatinine, uric acid, calcium).

**Fig. 2:**
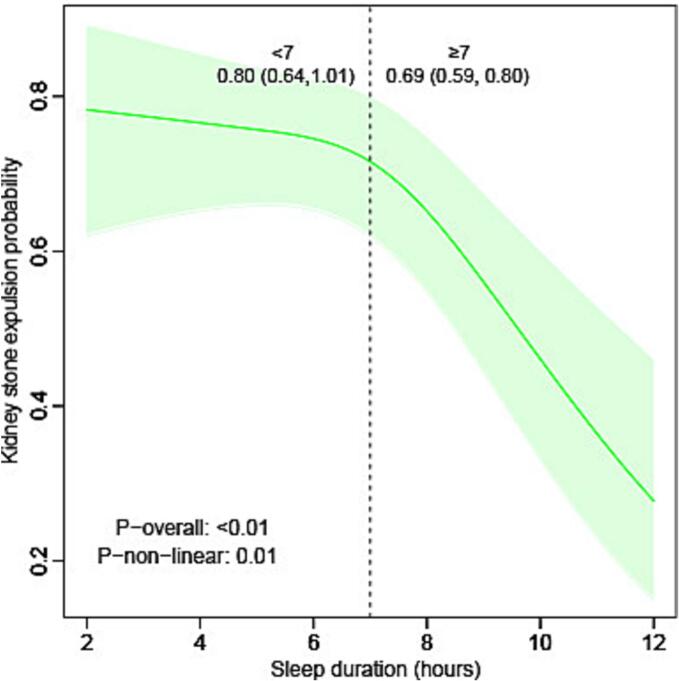
Restricted cubic spline plots illustrating the relationship between sleep duration on weekdays or workdays and kidney stone expulsion. Adjusted for age group, sex, race, metabolic equivalent, kilocalorie, marital status, educational level, the ratio of family income to poverty, alcohol, smoke, body mass index status, hypertension, diabetes mellitus, coronary heart disease, hyperlipidemia, estimated glomerular filtration rate, creatinine, uric acid, calcium. P value was calculated by likelihood ratio test.

**Supplementary Figure. 1:**
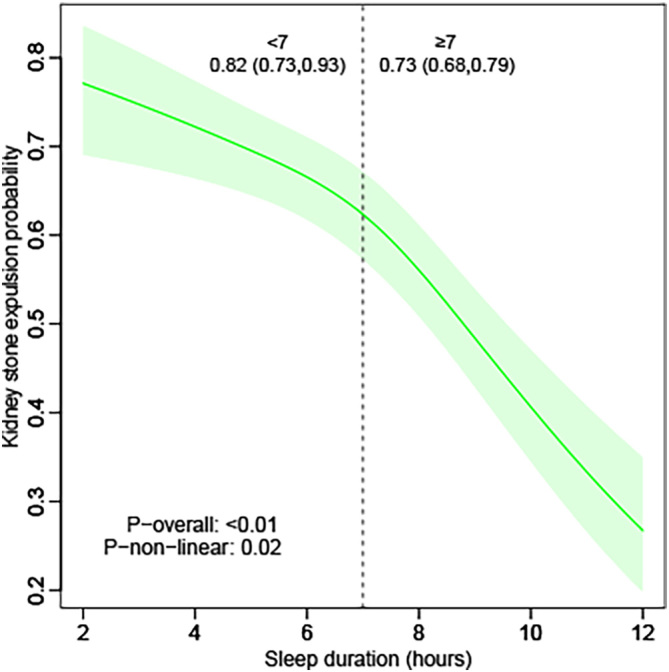
Restricted cubic spline plots illustrating the relationship between sleep duration on weekdays or workdays and kidney stone expulsion. Adjusted for age group, sex, race, metabolic equivalent, kilocalorie, marital status, educational level, the ratio of family income to poverty, alcohol, smoke, body mass index status, hypertension, diabetes mellitus, coronary heart disease, hyperlipidemia, estimated glomerular filtration rate, creatinine, uric acid, calcium. P value was calculated by likelihood ratio test.

The results of the RCS analysis (Fig. 2) further demonstrated a non-linear relationship between SDW and KSE (p = 0.01). In the fully adjusted model, the odds of KSE decreased sharply with SDW beyond seven hours (OR: 0.69 [0.59, 0.80]). Similarly, the RCS results after multiple interpolation of missing values of covariates are consistent with our initial analysis (Supplementary Fig. 1), again confirming the robustness of our conclusions.

## 4 Discussion

This research aimed to clarify the association between sleep duration on weekdays or workdays and kidney stone expulsion in adults with kidney stones using NHANES data from 2007 to March 2020 pre-pandemic. Our results suggested that participants with increased SDW had decreased rates of KSE. Although we observed some nonlinear associations between SDW and KSE, the trends were consistent across multivariable logistic regression models, providing new insights into the potential role of sleep health in kidney stone prevention and management.

Sleep plays a critical role in overall health, influencing numerous physiological processes such as metabolism, immune function, and hormonal regulation (Nguyen et al., 2024; Paunio, 2012; Wang et al., 2022). Short sleep duration has been associated with adverse health outcomes, including obesity, diabetes, cardiovascular disease, and increased all-cause mortality (Lange et al., 2024; Li et al., 2024). Conversely, prolonged sleep duration has also been linked to negative outcomes, such as increased mortality risk (Chaput et al., 2022). These findings suggest the existence of an optimal sleep duration for maintaining health. Our study hypothesizes that sleep duration may influence kidney stone expulsion through its effects on renal endocrine function, including water and electrolyte balance, and other systemic processes.

Our findings revealed a significant decrease in the likelihood of KSE among individuals who slept more than seven hours on weekdays or workdays. While the influence of SDW on KSE varied across different populations, the correlation remained significant after multivariate adjustment, suggesting that short sleep duration may serve as an independent risk factor in the conservative management of kidney stones.

Previous studies have highlighted the importance of optimal sleep duration. For example, Svensson et al. conducted a 20-year follow-up study in East Asia, demonstrating that both persistent short and excessive sleep durations were associated with increased mortality, with seven hours identified as the "optimal sleep duration" (Svensson et al., 2021). Similarly, data from the UK Biobank revealed a linear correlation between sleep duration and key indicators such as genetic and cognitive factors, brain structure, and physical health, with seven hours being optimal (Li et al., 2022). Another study involving 90,398 participants found that sleep durations below seven hours or above eight hours were associated with significantly increased mortality from cardiovascular and cerebrovascular diseases, cancer, and other causes (Liang et al., 2023). These findings align with our study, where the optimal SDW for reducing KSE rates was observed to be around seven hours. In our logistic regression models and restricted cubic spline analysis, participants with SDW beyond seven hours exhibited a significant decrease in KSE rates, particularly when SDW beyond nine hours.

Initially, we hypothesized that reduced sleep duration might increase physical activity, potentially aiding in the mechanical expulsion of kidney stones. However, our data indicated that participants with a history of kidney stone expulsion had lower MET levels. This finding aligns with a large prospective cohort study that found no independent relationship between physical activity and kidney stone formation (Ferraro et al., 2015). After adjusting for covariates such as MET, caloric intake, and body mass index, the negative association between prolonged SDW and KSE rates persisted, suggesting the involvement of other mechanisms. One plausible mechanism is related to the nocturnal regulation of renal function. During sleep, decreases in blood pressure and sodium excretion result in reduced nocturnal urine flow rates (Staessen et al., 1991; Shafiee et al., 2016). Longer sleep duration may further reduce nocturnal urine flow, thereby reducing the "flushing" effect necessary for spontaneous stone passage. In addition, circadian rhythmicity and endocrine changes during sleep - particularly variations in antidiuretic hormone (ADH) secretion (Mahler et al., 2013) - significantly affect urine concentration and flow. Increased ADH secretion during prolonged sleep increases urine osmolality and decreases urine volume, conditions that can adversely affect stone passage and even promote stone formation (Kiyohide et al., 2011). These hypothesis highlights the need for further basic and clinical research to explore the interplay between sleep duration, renal endocrine function, and kidney stone expulsion.

Our study suggests that optimizing SDW could serve as a noninvasive strategy for improving kidney stone expulsion rates as part of conservative treatment plans. The large sample size of this study provided robust statistical power. However, several limitations should be noted. First, It should be acknowledged that due to the cross-sectional design of this study, our results show associations rather than causal relationships between SDW and KSE. Further prospective or longitudinal studies are needed to establish causality. Second, the NHANES data on SDW and KSE were self-reported, which may introduce recall bias or misclassification. This could potentially affect the observed associations and should be taken into account when interpreting our results. Additionally, we could not account for other potential confounders, such as genetic predisposition, hydration levels, dietary intake, and environmental exposures. Future studies should incorporate objective sleep assessment methods, detailed kidney stone history records, and longitudinal designs to further validate the relationship between sleep duration and KSE rates. Finally, as this study was conducted on U.S. adults, the findings may not be generalizable to other populations.

In summary, our findings demonstrate a significant association between longer weekdays/workdays sleep duration and a decreased risk of kidney stone expulsion, underscoring the potential for non-invasive strategies targeting sleep behaviors in the management of kidney stones. Clinicians should consider sleep duration as part of comprehensive kidney stone management, while public health policymakers should emphasize the importance of proper sleep in the general population. Further research is warranted to elucidate the biological mechanisms underlying the relationship between sleep duration and both the formation and expulsion of kidney stones. Additionally, although our study focused on the dimension of sleep duration, it is important to note that sleep quality may also significantly affect kidney stone risk and expulsion, as poor sleep quality could contribute to metabolic and endocrine disturbances implicated in urolithiasis. Therefore, future research should explore whether addressing sleep quality, in addition to duration, can further improve outcomes for kidney stone patients.

## 5 Conclusion

In this nationally representative sample, longer SDW was associated with a reduced likelihood of KSE. However, given the cross-sectional nature of our study, this association does not imply causality. Further experimental and longitudinal research is needed to elucidate the causal pathways and underlying mechanisms linking SDW and KSE.

The following are the supplementary data related to this article.

Supplementary Table 1 Sensitivity Analysis of Baseline Characteristics between Included and Excluded Participants from the National Health and Nutrition Examination Survey dataset (2007–2020). Supplementary material 1

Supplementary Table 2 Association of sleep duration on weekdays or workdays with kidney stone expulsion, National Health and Nutrition Examination Survey dataset (2007–2020). Supplementary material 2

## CRediT authorship contribution statement

**Shuchao Ye:** Writing – review & editing, Writing – original draft, Visualization, Validation, Supervision, Software, Resources, Project administration, Methodology, Investigation, Funding acquisition, Formal analysis, Data curation, Conceptualization. **Dongming Lu:** Writing – review & editing, Funding acquisition, Formal analysis. **Damei Ye:** Writing – review & editing, Software, Funding acquisition, Formal analysis. **Bingyong You:** Writing – review & editing, Formal analysis. **Yongyang Wu:** Writing – review & editing, Validation, Formal analysis. **Shangfan Liao:** Writing – review & editing, Writing – original draft, Validation, Software, Resources, Methodology, Investigation, Formal analysis, Conceptualization.

## Ethics statement

This study was approved by the ethics review board of the National Center for Heath Statistics and written informed consents were obtained from each participant.

## Funding information

This study was supported by funding from Startup Fund for scientific research, Fujian Medical University [2019QH1258, 2022QH1255], Fujian Provincial Health and Health Youth Research Project [2022QN01010103] and Fujian Province Health and Youth Backbone Talent Training Project [2022GGA056].

## Declaration of competing interest

The authors declare that they have no conflicts of interest, either financial or personal, that could have affected the research presented in this manuscript.

## Data Availability

Data will be made available on request.
